# Ion Beam Modification of Carbon Nanotube Yarn in Air and Vacuum

**DOI:** 10.3390/ma10080860

**Published:** 2017-07-27

**Authors:** Jonathan G. Gigax, Philip D. Bradford, Lin Shao

**Affiliations:** 1 Department of Nuclear Engineering, Texas A&M University, College Station, TX 77843, USA; gigaxj@tamu.edu; 2 Department of Textile Engineering, Chemistry and Science, North Carolina State University, Raleigh, NC 27695, USA; philip_bradford@ncsu.edu

**Keywords:** ion-solid interaction, carbon nanotubes, ion beam welding, carbon nanotube yarn

## Abstract

We studied the effects ion beam irradiation on carbon nanotube (CNT) yarns. CNT yarn was fabricated by drawing and spinning CNT sheets from a vertically aligned CNT forest. The yarn was irradiated by 2.5 MeV protons in either vacuum or air. Irradiation in air was achieved by directing the proton beam through a 0.025 mm thick Ti window. Irradiation in vacuum occurred at a pressure of <10^−6^ torr at room temperature and at an elevated temperature of 600 °C. Tensile testing revealed that CNT yarn irradiated in air increased in tensile strength with increasing proton fluence. For yarn irradiated in vacuum, however, the strength decreased with increasing fluence. We believe that irradiation-induced excitation and trapping/bonding of gas atoms between tubes may play a role for the mechanical property changes.

## 1. Introduction

Among various structural formats of low dimensional carbon systems, carbon nanotube (CNT) yarns are unique because they represent an assembly of very long CNTs with a defined fiber structure. With the right CNT array growth conditions, these yarns can be easily fabricated through pulling and spinning of CNT bundles from an aligned CNT sheet [1,2]. The continuous linking of CNTs is realized through van der Waals bonding between tubes. However, the realistic applications of CNT yarns are limited because the CNTs are of finite length and there are only weak interactions between tubes. Recently, it was found that ion irradiation can be used to create displacements as inter-tube bridges to improve thermal properties of buckypaper [3,4,5,6]. Ideally, if ion irradiation can induce ‘welding’ in the yarn, then significant thermal, mechanical, and electric property changes are expected. Previous studies have shown that electron irradiation can induce welding of two tubes, provided they are in close proximity [7,8]. On the other hand, radiation damage, such as point defects and defect clusters, lead to property degradation. Amorphization occurs if CNTs are exposed to high dose irradiation [9]. Systematic studies are needed to optimize irradiation parameters. Previously, we have reported slight tensile strength changes for room temperature irradiation of yarns in air [10]. In the present study, the irradiation is increased to a higher dose and a comparison is made between irradiation in air at room temperature and vacuum at room temperature and high temperature. 

## 2. Experimental Procedure

In this study, we compare the effects of ion irradiation of CNT yarns in air and in vacuum. The CNT yarn was fabricated by drawing from an aligned CNT forest on a glass substrate. The typical drawing speed is ~2.5 mm/s. At the same time, the yarn was spun to improve integrity and density, yielding yarn with a diameter of ~40 μm and a twist angle of 30°. The as-fabricated CNT yarn was irradiated by a 2.5 MeV proton beam under three conditions with a flux of 2.5 × 10^12^ cm^−2^ s^−1^. For the irradiation in vacuum, two sets of yarn irradiations were performed. For the first set, the yarn was irradiated at room temperature to fluences ranging from 1 × 10^13^ to 1 × 10^15^ cm^−2^. In the second set, the yarn was heated to 600 °C and irradiated to doses ranging from 1 × 10^14^ to 1 × 10^16^ cm^−2^. The vacuum of the irradiation chamber was better than 10^−6^ torr. For irradiation in air, the proton beam was transmitted through a 25 μm Ti window into air. The energy losses of the proton beam were calculated using Stopping Range of Ions in Matter (SRIM 2013) [11]. The total energy loss of the proton beam after passing through the Ti window was about 1 MeV, resulting in protons with an energy of 1.5 MeV. At this energy, the protons are able to penetrate several centimeters in air. Since the yarn was positioned very close to the Ti window with less than 5 mm of an air gap, no significant beam dispersion is expected, with minor energy losses (250 keV). The yarn was irradiated to fluences from 5 × 10^12^ to 1 × 10^16^ cm^−2^ which were calculated by using the dose measurements in the vacuum chamber. Since the beam spot is about 6.5 mm × 6.5 mm, each yarn is irradiated three times (non-overlapping) to create a continuously irradiated region larger than 1 cm in length to facilitate the subsequent tensile tests. 

CNT yarns were characterized using a TESCAN LYRA focused ion beam (FIB)/scanning electron microscope (SEM) (TESCAN, Brno, Czech Republic) operated at 10 kV for electron beam imaging. A focused ion beam with an energy of 30 keV was used to cut the yarn for cross-sectional imaging. The tensile tests were conducted on a custom tensile tester operated at a strain rate of 0.2 mm/min. A DC motor (Maxon EC-MAX, Maxon Motor, Sachseln, Switzerland) and a worm gear assembly with a 1300:1 gear ratio were used to drive the crossheads of the tensile tester apart. A 200 N load cell (Futek FSH00097, FUTEK Advanced Sensor Technology, Irvine, CA, USA) and a linear potentiometer (TT Electronics, Fullerton, CA, USA) were utilized to measure the force and displacement, respectively. For good statistics, more than 10 CNT yarn pieces were characterized for each irradiation condition, with the exception of the yarn irradiated in vacuum at room temperature. For this set, only a few yarns were irradiated at each dose. 

## 3. Results and Discussion

**Figure 1:**
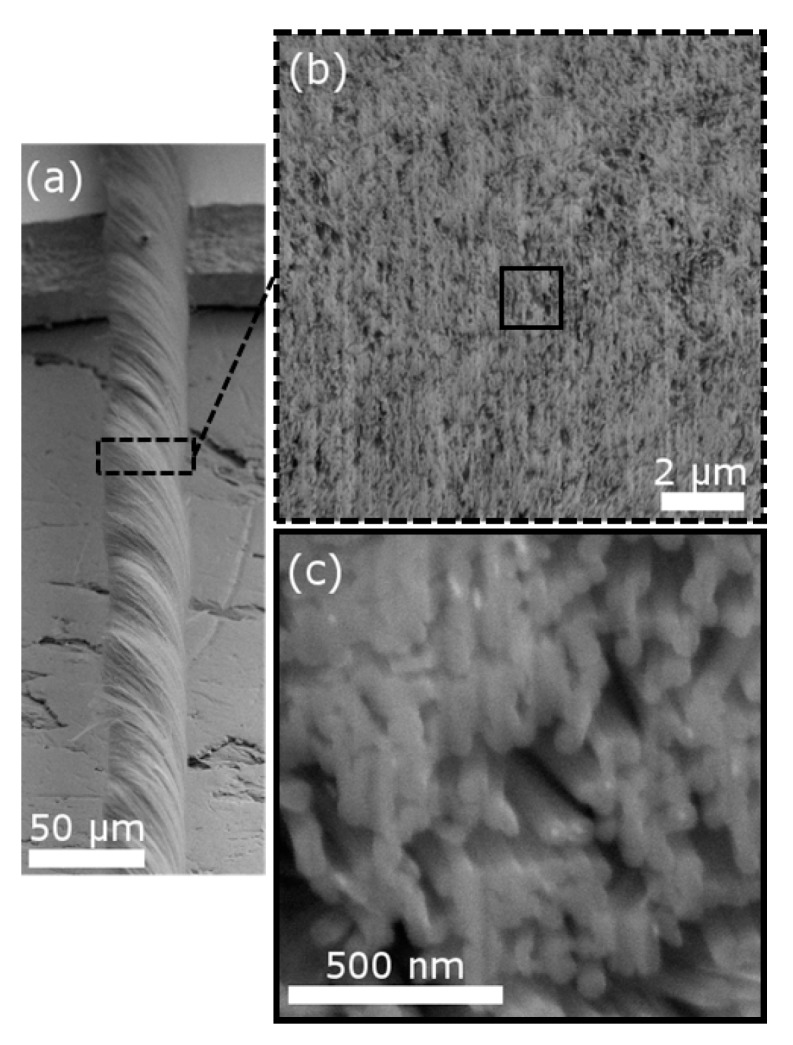
(**a**) Low magnification SEM micrograph of CNT yarn prior to ion milling and (**b**) cross-section image of the CNT yarn. The dashed box in (**a**) highlights the region used for cross-section imaging and a solid black box in (**b**) indicates a region used to obtain high magnification images in (**c**).

Figure 1a shows a typical image of a CNT yarn fiber. The fabricated yarn was about 40 μm in diameter. The diameter can be controlled by adjusting the spinning and drawing rate, as well as the amount drawn from the CNT forest. However, for 1.5 MeV and 2.5 MeV protons, the projected range in the CNT yarn is about 60 μm and 100 μm according to SRIM calculations. Hence, the yarn diameter must be less than 60 μm in order to uniformly modify the entire yarn without implanting H in the material. Figure 1b shows a typical cross-section of CNT yarn. Porous regions are clearly visible. Under higher magnification (Figure 1c), it can be seen that many tubes are closely touching each other. These tubes are potential sites for radiation-induced tube linking. The porous regions are shown to be due to misaligned CNTs and bundles from the drawing and spinning process.

**Figure 2:**
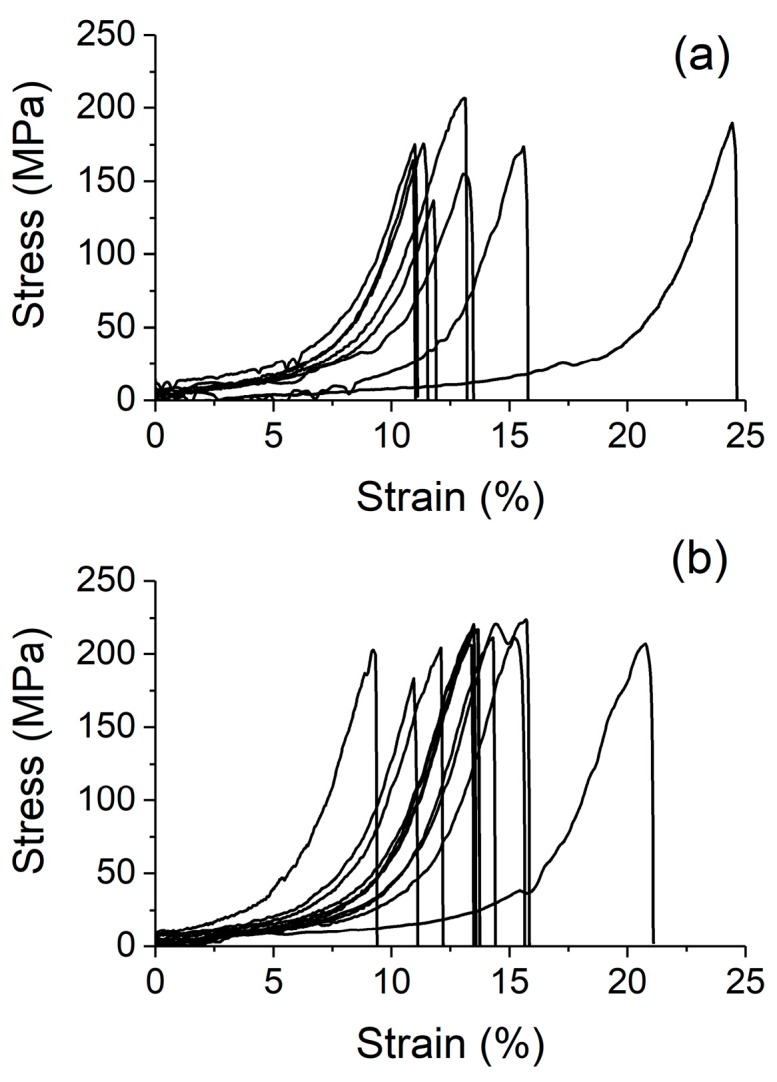
Stress–strain curves of (**a**) unirradiated CNT yarn and (**b**) yarn irradiated to 1 × 10^16^ cm^−2^ in air.

Figure 2a,b show typical, uncorrected stress–strain curves obtained from unirradiated yarn and yarn irradiated to 1 × 10^16^ cm^−2^ in air, respectively. Although a large spread of mechanical responses were observed, the tensile strength for both the unirradiated and irradiated yarn varied less than ~15%. The yarn irradiated to 1 × 10^16^ cm^−2^ showed an overall higher tensile strength than the unirradiated yarn. However, the strain at which fracture was observed for the yarns exhibited a much larger variation. Closer inspection revealed that a majority of the yarn tested fractured at a similar strain, with the irradiated yarn showing a slightly higher average fracture strain than the unirradiated yarn. It is worth noting that there is very little, if any, plastic deformation exhibited by the specimens before and after irradiation.

**Figure 3:**
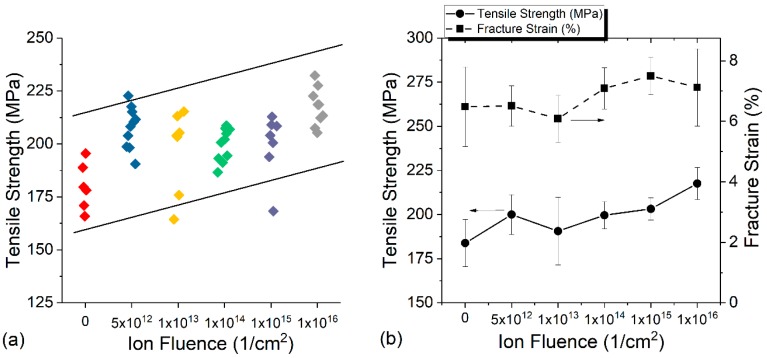
(**a**) Tensile strength comparison as a function of fluence and (**b**) comparison of tensile strength and fracture strain as a function of ion fluence for yarn irradiated in air.

Figure 3a compares the tensile strength of yarns after proton irradiation in air up to 1 × 10^16^ cm^−2^. For each irradiation fluence, at least six yarn pieces are shown for comparison. Despite scatter in the data, a trend from the tested yarn is clear. Figure 3b plots both the tensile strength changes and fracture strain changes as a function of dose. Statistically, there is a noticeable increase in tensile strength with increasing proton doses. Although the fracture strain appears to increase at the highest fluence, the large error bars do not permit a definitive conclusion on the strain changes. 

**Figure 4:**
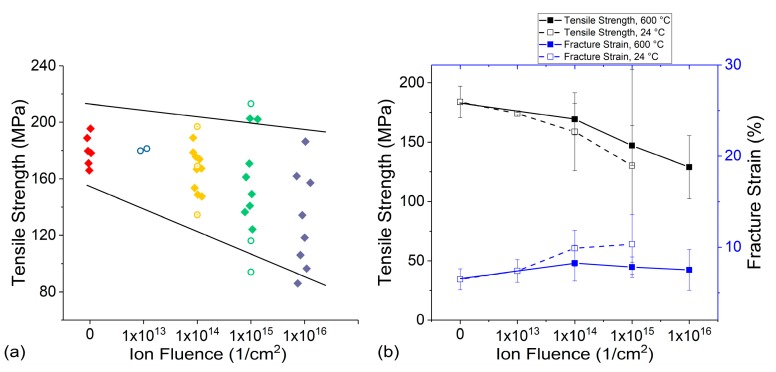
(**a**) Tensile strength comparison as a function of fluence and (**b**) comparison of tensile strength and fracture strain as a function of ion fluence for yarn irradiated in vacuum. Open circles and filled diamonds in (**a**) are for room temperature and high temperature irradiations, respectively.

Figure 4a summarizes the tensile strength changes for yarns irradiated in vacuum. Contrary to irradiation in air, the tensile strength systematically decreased with increasing proton fluences for both yarn irradiated at room temperature and at 600 °C. Figure 4b compares both tensile strength and fracture strain changes with error bars. It is clear that the tensile strength decreases with increasing proton doses. However, fracture strain, as with the in-air irradiation, does not exhibit a definitive trend. 

The tensile strength, the resistance of a material to breaking under tension, is sensitive to inter-tube bonding. Previous studies have shown that, for multilayered graphene, inter-plane carbon displacements can act as a seed atom to pull atoms from the graphene plane and form a carbon atom single chain to enhance friction during graphene gliding [12]. Similar carbon chain-like defects have been observed when two nanotubes are gliding on each other, if there is a carbon interstitial trapped between the tubes. These mechanisms can lead to enhanced gliding resistance and increased tensile strength. On the other hand, the final fracture strain is largely determined by the fracture toughness of tubes themselves and is insensitive to small or moderate amounts of inter-tube crosslinking. 

**Figure 5:**
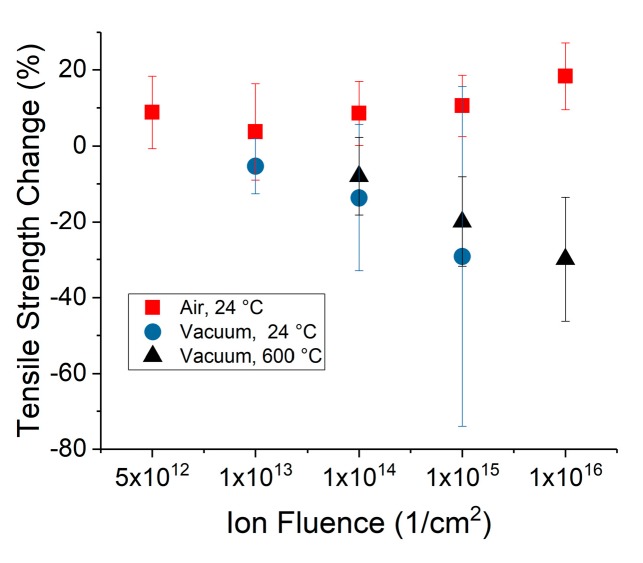
Comparison of tensile strength change as a function of fluence for yarn irradiated in air and in vacuum.

Figure 5 compares the change in tensile strength as a function of ion fluence for the yarn irradiated in air and vacuum. At the highest fluence, the yarn exhibited an increase in tensile strength of 18 ± 8.8% for the in-air irradiation. However, the yarn irradiated in vacuum showed up to a 30 ± 16% decrease in strength at 600 °C and 30 ± 44% decrease at 24 °C, albeit at a lower dose. The improvement in tensile strength after irradiation in air compared to the significant decrease in strength after irradiation in vacuum demonstrates the importance of the irradiation environment on mechanical property changes. It is also important to note that the displacement rate of a 1.5 MeV proton is approximately twice that of a 2.5 MeV proton. This difference in displacement rate does not significantly change the behavior as the fluences chosen span several decades with significant overlap. On the other hand, the damage-induced by the primary ion beam is lower in vacuum, and results in an even more dramatic difference in behavior between CNT yarn irradiated in air and in vacuum.

Reinforcement of CNTs by electron-induced inter-tube bridging has been previously reported, with a 30-fold increase of the bending modulus. In conjunction with modeling studies, Kis et al. have shown that electron irradiation-induced carbon displacements can form new bonds between two neighboring tubes and increase sliding resistance upon loading [9]. In order to form such C interstitial mediated bonding, it is required that two neighboring tubes be closely positioned to each other. In one stable configuration identified, a carbon interstitial forms a four-fold coordinated bond, with the top two bonding at 60 degrees between them and the bottom two bonding at 88 degrees between them [9]. If two tubes are too far apart, this type of inter-tube bonding cannot form. On the other hand, it was suggested that other species such as oxygen and nitrogen molecules absorbed from air, or hydroxyl and carboxyl groups from acid purification process can also lead to crosslinking. Different from carbon mediated linking, the linking through carboxyl functional groups can occur at larger separation distances. Additional evidence of this is supported by a comparison study between CNT yarn irradiated by gamma radiation in air and vacuum, and from irradiation in low earth orbit [13]. It should be noted that, since gamma radiation does not induce displacements, significant degradation from structural damage of the CNTs is not expected.

Exposure to environment in low earth orbit, which features a combination of UV, high energy ionizing radiation (protons and electrons), and atomic oxygen was shown to influence mechanical properties of the yarn [14]. The fracture strain of the CNT yarns exposed to this environment decreased, with or without an accompanying decrease in the tensile strength (dependent on exposure conditions), and was thought to arise from the cross-linking of CNTs shown in a previous study [15]. In our study, the fracture strain does not show a clear trend, unlike the improvement in tensile strength, suggesting that the number of cross-links formed provide better load-sharing between CNTs without embrittling the material. It is important to note that the exposure time of the CNT yarn irradiated in this study was several orders of magnitude less than that performed in low earth orbit and other effects, such as surface erosion and amorphization, were not observed in CNT yarns irradiated by protons in air.

We believe that irradiation in air has two additional contributions to improvements in the tensile strength of yarns. First, irradiation-induced ionization and dissociation of air molecules increases the chance for interaction with carbon atoms on yarns, particularly for sites with dangling bonds. Second, involvement of air molecules in the bridging process reduces the need to have an extremely short separation distance. Despite the increasing defect level in CNTs from irradiation, the yarn showed improvements in tensile strength [10]. Therefore, it is expected that tensile strength can be increased for irradiation in air due to crosslinking assisted load transfer that outpaces CNT degradation. 

For irradiation in vacuum, the crosslinking is limited to a carbon interstitial-mediated mechanism, and occurs for tubes that are very closely together. We note that, unlike previous studies, to purposefully implant H into the CNTs, the proton beam completely penetrates the CNT yarn without doping, thus no crosslinks from H implantation are expected [16]. The lower efficiency in creating crosslinks results in an overall degradation in mechanical strength arising from the defects within tubes dominating the overall property changes. That is, without crosslinks, vacancy defects produced by displacements within the tubes reduce the mechanical strength. Despite irradiating the yarn at 600 °C to improve interstitial-vacancy recombination, the residual defects surviving from the recombination process play a significant role in property degradation.

Although the reported mechanical property improvement is small for in-air irradiation, the present study shows promise to further increase tensile strength of CNT yarns. One method for improvement is to further increase doses, and the other is to create environments that facilitates atom/molecule trapping. However, adding additional fluence may not be a realistic approach due to the high throughput required for various applications. As for the second approach, molecules and functional groups can be intentionally introduced during the pre-irradiation preparation stages to maximize the inter-tube trapping. 

## 4. Conclusions

Through a comparison study between CNT yarn irradiated in air and vacuum, we found that the tensile strength of yarns can be enhanced with increasing proton fluence if the irradiation is performed in air. In direct contrast to this behavior, the tensile strength was observed to decrease dramatically for irradiation in vacuum. The study suggests that air molecules play an important role in creating inter-tube crosslinking under ion irradiation and increase structural integrity under an external load. Without sufficient crosslinking, the overall mechanical property is continuously degraded at higher proton doses due to increasing defect levels within tubes arising from atom displacements.
